# Epicardial Automatic Implantable Cardiac Defibrillator In A Child With Symptomatic Bugada Syndrome

**Published:** 2011-07-03

**Authors:** Jose M Moltedo, Mauricio Abello, Sivori Gustavo, Celada Javier, Pablo Garcia Delucis

**Affiliations:** 1 Electrophysiology Section, FLENI, Buenos Aires, Argentina; 2 Section of Pediatric Cardiovascular Surgery, FLENI, Buenos Aires, Argentina

**Keywords:** Brugada, Pediatrics, Automatic Implantable Defibrillator

## Abstract

An 18 month old 14 kg male with symptomatic Brugada syndrome underwent placement of an epicardial automatic implantable cardiac defibrillator using a single coil transvenous lead sutured to the anterolateral aspect of the left ventricle.

## Introduction

Brugada Syndrome is a known cause of sudden cardiac death in young individuals without structural heart disease. The most valid therapy for symptomatic individuals is the use of an automatic implantable cardiac desfibrillator (AICD). Despite technological advances which have allowed the use of AICDs in children, there are still technical limitations in smaller individuals. Innovative implant approaches have been described in the literature [1,2]. However, there is paucity of data regarding AICDs implants in small pediatric patients with Brugada syndrome [3]. This case constitutes another example of the feasibility of using a transvenous catheters sutured to the epicardium in small pediatric patients.

## Case Report

**Figure 1:**
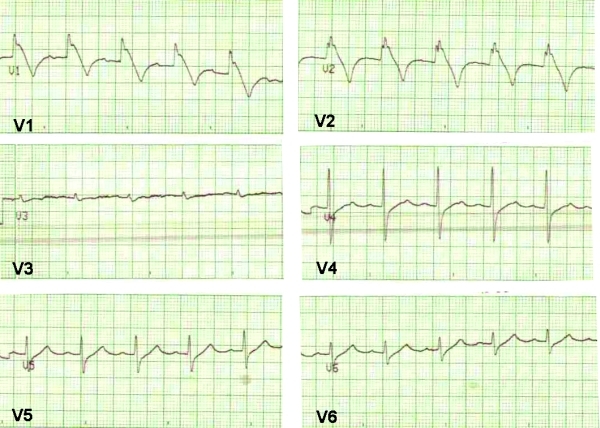
Precordial leads showing sinus rhythm and a type I Brugada pattern.

An 18 month-old previously healthy male presented to the emergency department with hemodynamically unstable ventricular tachycardia. His father had a diagnosis of asymptomatic Brugada syndrome. Tachycardia terminated spontaneously before external defibrillation was attempted. His baseline ECG showed a coved type Brugada syndrome pattern (Figure 1) and the implantation of an AICD was decided.

**Figure 2:**
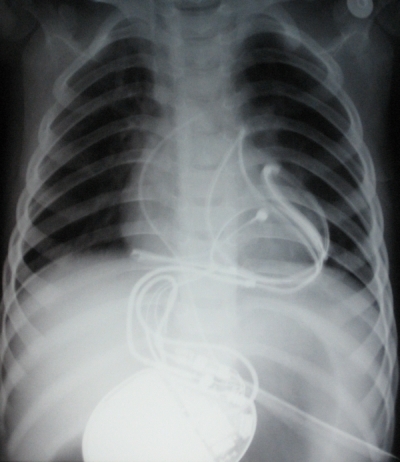
AP chest and abdominal X ray showing the leads and the generator position. The single coil transvenous defibrillator lead is sutured to the postero-lateral aspect of the left ventricle.

Due to his weight (14 kg), an epicardial approach was elected over a transvenous one. A standard transvenous single coil lead (GUIDANT Endotak Reliance SG SN 0180-102758) was sutured to the posterolateral epicardial aspect of the left ventricle, avoiding the left anterior descending coronary artery. An epicardial bipolar steroid eluting passive fixation catheter (Medtronic Capsure Epi Model 4968) was sutured to the right ventricular epicardium for sensing and pacing. Leads were tunneled to the abdomen and the AICD device (GUIDANT VITALITYTM REF 0180 VR) was placed in a sub rectus pocket in the right hypochondrium (Figure 2). Ventricular fibrillation was induced with a T wave shock and it was successfully recognized by the device and terminated with 20 Joules.

The patient was admitted to the pediatric cardiovascular recovery unit and had an uneventful postoperative course until he was discharged, 2 days following the implant.

## Discussion

AICDs remain the only recognized effective therapy for sudden death prevention in symptomatic Brugada syndrome patients. Difficulties arise with smaller pediatric patients due to the leads and defibrillators sizes. Concerns are related to the development of venous occlusions and to local complications like wound dehiscence or skin erosion.

The alternatives for small patients are not optimal. Pericardial patches can be used, but there are limitations related to the potential development of severe complications, including constrictive pericarditis or postpericardiotomy syndrome. There are previous reports of different innovative alternatives for pediatric patients requiring AICDs using transvenous or subcutaneous coils in non-traditional positions, hence avoiding the occurrence of long term vascular complications [1,2,4]. However, with subcutaneous coils the potential for coil damage or migration exists and has been described as a complication to this approach [2].

In our patient a single coil transvenous catheter sutured to the epicardium was used. This technique has been previously described in a study by Cannon et al [1]. Due to the potential for cardiac strangulation with the lead secondary to cardiac somatic growth, the great vessels and the coronary arteries were carefully avoided. The lead was placed in the posterolateral aspect of the left ventricle, laterally to the left anterior descending coronary artery. In addition, with this lead position, an adequate defibrillation vector was obtained by placing the generator in a pocket created in the left hypochondrium (Figure 2). Defibrillation thresholds at implant have been described to be adequate with these novel techniques for AICD placements.  Cannon and co-workers [1] postulated that AICDs should be tested after chest closure with sternal wires, due to the fact that air is a poor conductor potentially affecting defibrillation thresholds. In our case it was tested before closure not affecting the results. Testing before closing the chest allows for the possibility of changing the position of the coil in case the threshold was high.

## Conclusion

The placement of a transvenous defibrillator lead in the pericardial aspect of the heart appears to be a good alternative for smaller patients with the need for an AICD. Its location in the lateral aspect of the left ventricle to the left of the left anterior descending coronary artery may be a good position to avoid cardiac strangulation and compromise of the coronary vasculature, and additionally providing a good defibrillation vector.
